# Risk of Editorial Bias: A Case Study of Factors Contributing to Review Time in a Leading Journal in Dentistry

**DOI:** 10.1002/cre2.70122

**Published:** 2025-03-30

**Authors:** Momen A. Atieh, Nabeel H. M. Alsabeeha

**Affiliations:** ^1^ Mohammed Bin Rashid University of Medicine and Health Sciences, Hamdan Bin Mohammed College of Dental Medicine, Dubai Healthcare City Dubai United Arab Emirates; ^2^ Sir John Walsh Research Institute, Faculty of Dentistry University of Otago Dunedin New Zealand; ^3^ School of Dentistry University of Jordan Amman Jordan; ^4^ Consultant Prosthodontist, Ras Al‐Khaimah Dental Centre Emirates Health Services United Arab Emirates

**Keywords:** publication delay, randomized controlled trial, review time, risk of bias

## Abstract

**Objectives:**

The aim of this study was to evaluate the risk of editorial bias in the field of Dentistry by examining surrogate measures which can be readily extracted from published randomized controlled trials (RCTs) in a journal of high impact factor.

**Material and Methods:**

RCTs published between January 2019 and March 2023 were manually downloaded. Data related to author affiliation, dates of submission and first publication, study location, review time, compliance with Consolidated Standards for Reporting Trials (CONSORT) checklist, ethics approval number, clinical trial registration time, reported outcomes, and eligibility criteria in registries and sample size calculation were extracted.

**Results:**

A total of 40 RCTs were included in this cross‐sectional study. The mean review time was 165.38 ± 91.40 days with 55% of RCTs exceeding 120‐day review time. A total of 23 RCTs (57.5%) were compliant with the CONSORT statement. The review time of RCTs with editorial co‐authorship was significantly shorter than the review time of RCTs that had no authors from the editorial team (91.75 ± 42.03 vs. 239.00 ± 63.00 days; *p* < 0.001).

**Conclusions:**

RCTs with editorial co‐authorship in the field of Dentistry were statistically favored in the initial screening or peer‐review process having significantly short review time.

**Practical Implications:**

Scientific journals should adopt a double‐blind peer‐review process that is thorough, fair, and transparent to improve the quality of published research. To address any concerns related to editorial co‐authorship, editors should explicitly explain the peer‐review process in a commentary added to the published paper.

## 1 Introduction

The Committee on Publication Ethics (COPE) refers to an editor as someone who works closely with co‐editors and members of the editorial board to ensure a smooth and transparent processing of high quality research content from the time of initial submission to publication (COPE 2016). The COPE guidelines on editorial independence explicitly state that accepting or rejecting research papers should be solely based on the paper's originality and relevance (COPE 2016). Nevertheless, editors still retain the power of rejecting manuscripts at the entry level, changing members of the editorial board, selecting reviewers, and interpreting reviewers' comments and decisions (Sarigöl et al. 2017). Therefore, editors, associate editors, and members of editorial boards may play an indirect role in the peer‐review process with potential bias that could impact their decision on accepting or rejecting manuscripts, including their own. Lange and Frensch (1999) demonstrated that it is common for the citation rates of editors to significantly increase in their own journals. Such preferential citation is often driven by editors, associate editors, members of editorial boards, and authors submitting their work to the editor's journal. In the latter instance, authors may attempt to leave a positive impression on the editor or editorial board members by citing their published work in the submitted manuscripts (Levy et al. 2014).

The review time of editorial board members' publications was further investigated in articles published in two information science journals between 2003 and 2019. The authors concluded that the editorial board members dominated the corresponding and first authorship with significantly shorter review times of their articles compared to authors who were non‐board members (Xu et al. 2021). An independent assessment of editorial bias is not always possible, particularly when the review process is not an open peer one. In fact, there are no tools available to assist independent researchers in detecting editorial biases or evaluating the transparency of the review process. Hence, surrogate measures are used as an alternative to assess the potential risk of editors and co‐editors favoring the publication of their research work in their own journal. These surrogate measures may include the use of manuscript review time, quality of published papers, and compliance with common standards of reporting research. For example, COPE emphasizes that the review process should be completed in a timely manner and that authors should not experience any unnecessary delays (COPE 2016). In this context, an excessively long review time can be detrimental to science advancement and academician career (Björk and Solomon 2013), while a notably shorter handling and review time could be interpreted as an indication of a potential risk of editorial favoritism.

Randomized controlled trials (RCTs) require adherence to stringent and rigorous ethical and reporting criteria. Therefore, published RCTs provide an opportunity to evaluate the editorial bias in clinical research. We hypothesize that the publication of RCTs of editorial team members in their own journal would be associated with significantly shorter review time, less adherence to known standards of reporting RCTs and reported information in the clinical registry and higher risk of bias compared to RCTs published in the same journal by authors who do not serve on the editorial board of that journal. The aim of the present study, therefore, was to evaluate the risk of editorial bias in a journal of high impact factor in the field of Dentistry by examining surrogate measures extracted from published RCTs.

## 2 Methods

### 2.1 Study Design

A cross‐sectional based study. The Strengthening the Reporting of Observational Studies in Epidemiology (STROBE) statement (von Elm et al. 2014) was followed in the design and reporting of the present study.

### 2.2 Data Source

To be eligible for inclusion, the study title had to have the words “randomized controlled trial” and published in a leading journal in Dentistry between January 2019 and March 2023. The selected journal ranked high in the category of Dentistry as per the Web of Science and provided the received and acceptance dates of every publication. Such information has allowed the calculation of the review time (i.e., primary outcome). The time frame between January 2019 and March 2023 was selected to have higher chances of including RCTs that were more compliant with the author guidelines of the journal and the standards of reporting RCTs as stated in the Consolidated Standards for Reporting Trials (CONSORT) statement (Schulz et al. 2010).

The prior analysis for this exploratory cross‐sectional study was based on detecting odds ratio of three for the effect of editorial co‐authorship (Luty et al. 2009). A sample size of 20 papers per group was calculated assuming 80% statistical power, 5% level of significance, 50% constant proportion of controls exposed and a ratio of case‐to‐control as 1:1. A total of 40 papers was regarded as a feasible sample that would achieve sufficient saturation for information to be representative of recently published RCTs.

Ethical approval was not required for this study.

### 2.3 Data Collection

All the RCTs, published between January 2019 and March 2023, were manually downloaded from the webpage of the journal and a total of 40 RCTs were selected from pool of RCTs using a randomly generated number. The random number was generated using computer‐based random number generator (www.random.org). Each RCT was thoroughly scrutinized and data related to the author affiliation, dates of submission and first publication, study location, review time, compliance with CONSORT checklist, and ethics approval number were collected. The information in the clinical trial registry including the date of participant enrollment, primary and secondary outcomes, and inclusion and exclusion criteria were also collected. RCTs were classified as prospectively registered if the date of registration coincides with or precedes the start date of RCTs while RCTs registered after the date of first enrolment were considered to be retrospectively registered. The sample size calculation was re‐assessed, and the quality of the selected RCTs was evaluated using the domains of the Cochrane Collaboration's Risk of Bias (RoB) 2 tool (Higgins et al. 2022). The list of editors, associate editors, and members of the editorial board was obtained from the official journal webpage.

Our key independent variable, editorial co‐authorship, was defined as having at least one author of the published paper serving as an editor‐in‐chief, associate editor or a member of the editorial board at the time of publication. Based on this variable, each selected RCT was then assigned to one of two groups; RCT with or without editorial co‐authorship.

### Outcome Measures (Appendix I)

Primary outcome

Review time, defined as the time between received (i.e. submission) and acceptance date.

Secondary outcomes

Compliance with the 25‐item CONSORT checklist.

Registration of clinical trial; prospective or retrospective.

Differences between the data presented in the clinical trial registry and the published paper.

Quality of RCT as assessed by the Cochrane Collaboration's RoB 2 tool.

Sample size calculation.

### 2.4 Statistical Analysis

All data were recorded in Excel spreadsheets and analyzed using statistical software (IBM Statistical Package for Social Sciences (SPSS) for windows, version 28.0. Armouk, NY: IBM Corp). Descriptive statistics were used to report study characteristics in mean values and standard deviations for continuous variables. Frequencies and percentages were used to summarize categorical variables. Independent *t*‐test for continuous variables and chi‐square test for categorical variables were used to compare between the two groups (with and without editorial co‐authorship). The equivalent non‐parametric tests were applied when normality assumptions were not satisfied. Logistic regression analysis was used to examine how review time could be impacted by different independent variables such as editorial co‐authorship, compliance with CONSORT checklist, and clinical trial registry information and quality of the study. A cut‐off point of 120 days of review time was selected based on median review times reported in medical journals (Mohanty et al. 2021; Stamm et al. 2007).

## 3 Results

A total of 40 RCTs published between January 2019 and March 2023 were selected and included in this study. The number of RCTs were almost equally distributed across the years except for 2023, where only four RCTs were included. Half of the RCTs were conducted in Europe and the mean review time was 165.38 ± 91.40 days with 55% of RCTs exceeding 120‐day review time. A total of 23 RCTs (57.5%) were compliant with the CONSORT statement and only one RCT did not provide an ethical approval number. Four RCTs did not report information on the clinical trial registration, and only 17 RCTs were prospectively registered. The differences between information reported in registries and published papers were clearly obvious, with 57.5% of RCTs having discrepancies in the reported primary and secondary outcomes and 45.0% of RCTs showing differences in reported inclusion and exclusion criteria. The majority of the RCTs (65%) were judged to be at low risk of bias as assessed by the Cochrane Collaboration's RoB 2 tool and the sample size calculation was based on the primary outcome in 75% of the RCTs.

The review time of RCTs with editorial co‐authorship was significantly shorter than the review time of RCTs that had no authors from the editorial team (91.75 ± 42.03 vs. 239.00 ± 63.00 days; *p* < 0.001). The review time of RCTs conducted in European locations was also shorter than those conducted in non‐European locations. However, the difference was not statistically significant (140.45 ± 79.64 vs. 190.30 ± 97.45 days; *p* = 0.09). Moreover, RCTs without editorial co‐authorship were more likely to exceed a 4‐month review time. A total of 18 RCTs without editorial co‐authorship had a review time of more than 120 days compared to only 4 RCTs with editorial co‐authorship (*p* < 0.001). In fact, none of the RCTs without editorial co‐authorship had a review time of less than 90 days. Another statistically significant difference was noted in terms of the similarities in the reported inclusion and exclusion criteria between clinical trial registry and published paper. RCTs with no editorial co‐authorship were more likely to adhere to the same inclusion and exclusion criteria stated in the clinical trial registry, with 65.0% of those RCTs demonstrating that similarity compared to only 35% of RCTs with editorial co‐authorship (*p* = 0.008). In terms of other studied variables, there were no significant differences between the two groups of RCTs (Table 1).

**Table 1:** Characteristics of selected studies with risk of editorial bias.

Variable	Editorial co‐authorship group (*n* = 20)	Noneditorial co‐authorship group (*n* = 20)	*p* value^a^	Relative risk (95% CI)^b^
Review time				
≤ 120 days	16	2	< 0.001	4.50 (1.85, 10.94)
> 120 days	4	18		
Study location				
European	12	8	0.21	2.25 (0.64, 7.97)
Non‐European	8	12		
Compliance with the CONSORT checklist				
Yes	14	9	0.11	2.85 (0.78, 10.47)
No	6	11		
Clinical trial registration				
Yes	18	18	1.00	1.00 (0.13, 7.89)
No	2	2		
Clinical trial registration^c^				
Prospective	9	8	0.74	1.25 (0.34, 4.64)
Retrospective	9	10		
Similarities between data presented in the published paper and clinical trial registry (primary and secondary outcomes)^c^				
Yes	7	6	0.73	1.27 (0.33, 4.98)
No	11	12		
Similarities between data presented in the published paper and clinical trial registry (inclusion and exclusion criteria)^c^				
Yes	5	13	0.008	2.60 (1.17, 5.78)
No	13	5		
Risk of bias^d^				
Low	14	12	0.51	1.56 (0.42, 5.76)
High	6	8		
Sample size calculation				
Yes	19	19	1.00	1.00 (0.06, 17.18)
No	1	1		
Was the sample size calculation based on primary outcome?^e^				
Yes	15	15	1.00	1.00 (0.21, 4.76)
No	4	4		

Abbreviation: CI, confidence interval.

^a^ Chi‐square test.

^b^ Computed for 2 × 2 tables.

^c^ Four randomized controlled trials did not report information on the clinical trial registration.

^d^ Cochrane Collaboration's risk of bias 2 tool.

^e^ The sample size calculation was not clearly stated to be based on the primary outcome in two randomized controlled trials.

When the review time was examined as an outcome variable, similar findings were noted. The explanatory variables that were significantly associated with a review time of more than 120 days were the editorial co‐authorship and similarity between clinical trial registry information and published paper in terms of reporting inclusion and exclusion criteria. RCTs with a review time greater than 120 days were more likely to be lacking authors who were members of the editorial team (*p* < 0.001) but were more likely to adhere to the same inclusion and exclusion criteria reported in the clinical trial registry (*p* = 0.04) compared to RCTs with editorial co‐authorship (Table 2).

**Table 2:** Characteristics of studies with review time of > 120 days.

Variables	*n* (%) review time > 120 days	*p* value^a^	Relative risk (95% CI)^b^
Co‐authorship by editorial team members			
Yes	4 (20.0)	< 0.001	4.50 (1.85, 10.94)
No	18 (90.0)		
Study location			
European	11 (55.0)	1.00	1.00 (0.57, 1.75)
Non‐European	11 (55.0)		
Compliance with CONSORT checklist			
Yes	10 (43.5)	0.09	3.12 (0.83, 11.79)
No	12 (70.6)		
Clinical trial registration			
Yes	20 (55.6)	0.83	1.11 (0.40, 3.09)
No	2 (50.0)		
Clinical trial registration^c^			
Prospective	9 (52.9)	0.77	1.22 (0.33, 4.57)
Retrospective	11 (57.9)		
Similarities between data presented in the published paper and clinical trial registry (primary and secondary outcomes)^c^			
Yes	7 (53.8)	0.88	1.11 (0.28, 4.37)
No	13 (56.5)		
Similarities between data presented in the published paper and clinical trial registry (inclusion and exclusion criteria)^c^			
Yes	13 (72.2)	0.04	1.86 (0.97, 3.54)
No	7 (38.9)		
Risk of bias^d^			
Low	14 (53.8)	0.84	1.14 (0.31, 4.23)
High	8 (57.1)		
Sample size calculation			
Yes	21 (55.3)	0.88	1.11 (0.27, 4.55)
No	1 (50.0)		
Was the sample size calculation based on primary outcome?^e^			
Yes	17 (56.7)	0.74	1.13 (0.53, 2.42)
No	4 (50.0)		

Abbreviation: CI, confidence interval.

^a^ Chi‐square test.

^b^ Computed for 2 × 2 tables.

^c^ Two randomized controlled trials did not report information on the clinical trial registration.

^d^ Cochrane Collaboration's risk of Bias 2 tool.

^e^ The sample size calculation was not clearly stated to be based on the primary outcome in one randomized controlled trial.

The binary logistic regression analysis of the review time showed that only one variable, the editorial co‐authorship (i.e., editorial bias), has reached statistical significance (Table 3). In the final model, the lack of editorial co‐authorship presented as a useful and independent predictor variable that significantly influenced the review time of RCTs. This variable did not contain a value of 1.00 and had low standard error, indicating a statistically stable model. The odds ratios showed that RCTs that did not include an author from the editorial team were 41 times more likely to take longer than 120 days to be published. However, the other variables (study location, compliance with the CONSORT checklist, clinical trial registry information, sample size calculation, and study quality) did not contribute to the model's prediction, as they were found to be nonsignificant. The Nagelkerke *R* square suggested that the present logistic model was moderately strong and showed that 57.6% of the variation in review time was explained by this model. The estimates of the logistic regression model, the adjusted odds ratios for the studied variables, and their 95% CIs are summarized in Table 3.

**Table 3:** Results of logistic regression analysis.

Predictor variable	Outcome: review time > 120 days (no = 0, yes = 1)			
	*b*‐coefficient (SE)	*z‐* value	*p* value	Odds ratio (95% CI)
Co‐authorship by editorial team members				
(no = 0, yes = 1)	3.73 (1.30)	2.87	0.004	41.41 (1.18, 6.28)
European study location				
(no = 0, yes = 1)	−1.09 (1.20)	−0.91	0.37	0.34 (−3.44, 1.26)
Compliance with CONSORT checklist				
(no = 0, yes = 1)	1.17 (1.23)	0.95	0.34	3.22 (−1.24, 3.58)
Prospective clinical trial registration				
(no = 0, yes = 1)	−0.10 (0.99)	−0.10	0.92	0.90 (−2.04, 1.84)
Low risk of bias				
(no = 0, yes = 1)	−0.56 (1.10)	−0.51	0.61	0.57 (−2.72, 1.60)
Published paper versus clinical trial registry: Similar primary and secondary outcomes				
(no = 0, yes = 1)	−0.09 (1.18)	−0.08	0.94	0.91 (−2.40, 2.22)
Published paper versus clinical trial registry: Similar inclusion and exclusion criteria				
(no = 0, yes = 1)	−0.45 (1.14)	−0.39	0.70	0.64 (−2.68, 1.78)

*Note:* Model: Chi‐square = 20.27, df = 7, *p* = 0.005.

Abbreviation: CI, confidence interval.

## 4 Discussion

The present study showed that RCTs, including authors from the editorial team (such as editors, associate editors, or members of the editorial board), had a significantly shorter review time and were more likely to report different inclusion and exclusion criteria than those published in the clinical trial registry compared to RCTs without editorial co‐authorship. There were no significant association between editorial co‐authorship and study location, compliance to CONSORT checklist, clinical trial registration (prospective vs. retrospective), risk of bias, sample size calculation, or differences between reported primary and secondary outcomes in the clinical trial registry and published paper.

Several studies (Luty et al. 2009; Scanff et al. 2021; Taşkın et al. 2022; Yegros and Amat 2009) examined the factors that influenced the review time of submitted manuscripts. Yegros and Amat (Yegros and Amat 2009) coined the term “editorial delay” to refer to the review time and found no correlation between the study location and editorial delay, which is in accordance with our results. The authors, however, showed a wide variance in the review time and concluded that such variance can be interpreted as an editorial bias but without directly measuring the effect of editorial co‐authorship. In contrast, Taskin and colleagues (Taşkın et al. 2022) directly examined the editorial co‐authorship, amongst other factors, that influenced the review time in six information science journals. The results showed that papers with editorial co‐authorship had significantly shorter review times than papers published by authors who were not members of the editorial board. The difference was more apparent in journals that followed a single‐blind review process where reviewers were still aware of the authors and their affiliations. Similarly, Scanff and co‐workers (2021) surveyed 5468 biomedical journals and showed that papers with editorial co‐authorship were more likely to be accepted and published within 3 weeks of submission. These authors raised the question of whether a meaningful, thorough and robust peer‐review process is being conducted for the published work of editors and associate editors in their own journals. In a retrospective study by Luty and colleagues (2009), data were collected from 20 leading medical journals in five medical specialties. The findings showed that journals are three times more likely to accept and publish papers submitted by their editorial team members compared to those submitted by their rivals. One interesting finding of the present study was the substantial proportion of RCTs that had discrepancies between the information published in the clinical trial registry and the final paper. Differences in the inclusion and exclusion criteria were specifically more likely to be noted in RCTs with editorial co‐authorship or those with a review time shorter than 120 days. Similar findings were also reported in other studies, (Anand et al. 2014; Charles et al. 2009; Huić et al. 2011) where such differences represented an obvious deviation from published author guidelines but did not appear to influence the editor's decision to accept or reject manuscripts (van Lent et al. 2015).

The association between editorial co‐authorship and an unusually shorter review time can be interpreted in two ways. One could assume that papers with editorial co‐authorship are expected to have shorter review times because members of the editorial team have the expertise and scientific merits and are more familiar with the journal's scope and author guidelines that speed‐up the review process of these papers. On the other hand, it could be argued that the significant difference in the review times between the two RCTs groups, with RCTs lacking editorial co‐authorship being 41 times more likely to exceed 120 days, may indicate a risk of editorial bias and a preferential review process. More so, the strong association between editorial co‐authorship and discrepancies in the inclusion and exclusion criteria reported in the clinical trial registry and those reported in the published paper might suggest an insufficient review or cross‐checking of information at the initial editorial screening, which again could be highly suggestive of risk of bias in the presence of editorial co‐authorship.

It has been long hypothesized that social position and affiliation of authors could influence reviewers' evaluation and proposed decisions, particularly in journals where no blinded or open peer review process exists (Crane 1967). In such instances, reviewers would be aware that the work being reviewed belongs to an editor, associate editor, and/or a member of an editorial board, and hence, would be more keen to expedite the review process with less critique that might delay its publication, or otherwise, hampers the future handling of the reviewer's own work in that journal as a repercussion. In a worldwide survey of 3040 academics, 70% of participants expressed the need for the implementation of a double‐blind peer review process to avoid potential bias related to reviewer's personal opinion of the author, institution, race, or country (Kmietowicz 2008). The reality, however, is that majority of leading journals in Dentistry remain adherent to the single‐blind review.

Other variables that could influence the review time include the authors' metrics and citation network. Specifically, the Hirsch index may offer valuable insights into an author's academic influence and reputation, potentially leading to a more favorable review process, especially in single‐blinded reviews where authors' identities are not anonymized (Frachtenberg and McConville 2022; Harris et al. 2017). In addition, gender and ethnicity are factors that may go unnoticed. Despite efforts by editors to remain objective, the publication output remains disproportionately skewed in favor of male authors (Squazzoni et al. 2021). Some studies suggest that the underrepresentation of women in roles such as editor‐in‐chief and on editorial boards could contribute to gender bias in the review process (Squazzoni et al. 2021; Drozdz and Ladomery 2024). Similarly, ethnic and geographic biases have been observed, with a low likelihood of accepting manuscripts from non‐English speaking countries in single‐blind peer reviews (Okike et al. 2016; Skopec et al. 2020). Double‐blind peer review has been shown to mitigate these biases, enhancing the likelihood of manuscripts from women and ethnic minorities being accepted (Budden et al. 2008; Snodgrass 2006). While authors' metrics were not included in this study due to challenges in capturing such data at the time of publication, the exclusion of factors like author metrics, ethnicity, and gender represents a limitation of the current study. Future research could explore these factors to provide a more comprehensive understanding of the determinants of review time.

It is a fundamental understanding that editors and associate editors are the gatekeepers of the journal who are responsible for maintaining a transparent, unbiased, and fair peer‐review mechanism. These roles are guarded by editorial independence, and therefore, all authors with editorial roles must have no conflict of interest in meeting the requirements of the International Committee of Medical Journal Editors (ICMJE) (Zarin et al. 2005). Additionally, they must have actually followed the author guidelines of their journal and, most importantly, have their work reviewed by independent experts in a rigorous and transparent manner that is accessible to other researchers to assess and evaluate (Shen et al. 2015). Moreover, quality assessment tools, such as the Cochrane Collaboration's RoB tool and Enhancing the Quality and Transparency of Health Research Network (EQUATOR) checklists should be revised to include a domain that specifically evaluates any potential editorial risk of bias by adding signaling questions such as: Is there any editorial co‐authorship? If yes, was the contribution of the editorial member clarified in the paper? Does the journal adopt a blinded or open peer‐review process? Furthermore, publishers of scientific journals may adopt a policy where editors and associate editors are not allowed to submit manuscripts in their own journals or have a clear and transparent mechanism on how the manuscripts of editorial board members are handled (Helgesson et al. 2022). For example, COPE recommends that any paper submitted with editorial co‐authorship should undergo an independent peer‐review process, and a commentary or a note should be added to describe how that paper was handled from the time of submission and through the review process till the decision of acceptance (COPE 2016).

## 5 Conclusion

Within the limitations of this study, the current findings showed that RCTs with editorial co‐authorship in the field of Dentistry were statistically favored in the initial screening or peer‐review process having significantly shorter review time and accepting more deviations from the reported information in the clinical registries compared to RCTs without editorial co‐authorship. Scientific journals should adopt a double‐blind peer‐review process that is thorough, fair, and transparent to improve the quality of published research.

## Author Contributions

**Momen A. Atieh:** concept/design, data collection, data analysis/interpretation, drafting article, critical revision of article, and approval of article. **Nabeel H. M. Alsabeeha:** drafting article, critical revision of article, and approval of article.

## Conflicts of Interest

The authors declare no conflicts of interest.

## Supporting information


**Supplementary Appendix I:** Variable definitions.

## Data Availability

The data that support the findings of this study are available from the corresponding author upon reasonable request.
