# Supplementary material for: Risk of Editorial Bias: A Case Study of Factors Contributing to Review Time in a Leading Journal in Dentistry
Source: Clin Exp Dent Res. 2025 Mar 30;11(1):e70122. doi: 10.1002/cre2.70122 (PMC11955181; doi:10.1002/cre2.70122)
Supplement: Supplementary file 1 — Supplementary Appendix I: Variable definitions. [file CRE2-11-e70122-s001.docx]

**Supplementary Appendix I:** Variable definitions

| **Variable** | **Definition** |
| --- | --- |
| Review time | The time between received and acceptance date of a paper |
| Study location | The physical location at which a study was conducted |
| Compliance with CONSORT checklist | The CONSORT statement consists of 25 items that provide guidance on presenting and reporting RCT |
| Clinical trial registration | Prospective clinical trial registration: It is a registration process whereby investigators report on trial aims and methods in a clinical trial registry platform before enrolling the participants.  Retrospective clinical trial registration: It is a registration process whereby investigators report on trial aims and methods in a clinical trial registry |
| Similarities between the published RCT and clinical trial registry | The similarities between the published RCT and clinical trial registry in terms of reporting primary and secondary outcomes and inclusion and exclusion criteria |
| Risk of bias | The Cochrane Collaboration’s risk of bias 2 tool was used to assess the quality of RCT. The tool is used to assess the following domains based on answers to signalling questions: bias arising from randomization process, bias due to deviations from intended interventions, bias due to missing outcome data, bias in measurement of the outcome and bias in selection of the repeated results. An overall judgement for each study is then derived as follows: (i) low risk of bias when the study was considered to show a low risk of bias, (ii) some concerns when few concerns were expected to be associated with the study in at least one domain, but not warranting categorization as a study with a high risk of bias for any domain, (iii) high risk of bias when the study was considered to be at high risk of bias in at least one domain or few concerns with regard to multiple domains were observed in the study such that these concerns significantly lowered confidence in the study results |
| Sample size calculation | The calculation of sample size determines the number of participants required to detect a clinically relevant treatment effect |

CONSORT: Consolidated Standards of Reporting Trials; RCT: randomized controlled trial
